# A comparison of the clinical characteristics and outcomes of aerobic and anaerobic prosthetic joint infections (PJIs): a single-center retrospective review

**DOI:** 10.5194/jbji-10-471-2025

**Published:** 2025-11-18

**Authors:** Lemuel R. Non, Poorani Sekar

**Affiliations:** 1 Division of Infectious Diseases, Department of Internal Medicine, University of Iowa Hospitals and Clinics, Iowa City, IA, USA

## Abstract

**Background**: Prosthetic joint infection (PJI) is an uncommon but serious complication of joint arthroplasty, associated with significant morbidity and healthcare costs. Anaerobic organisms are an under-recognized cause of PJI, either as sole pathogens or within polymicrobial infections, and data on their clinical impact are limited. This study compared clinical presentation and outcomes of anaerobic vs. aerobic PJIs. **Methods**: This is a retrospective review of 284 patients who met Musculoskeletal Infection Society (MSIS) criteria for PJI from 2014 to 2020 at the University of Iowa Hospitals and Clinics (UIHC). A total of 38 had anaerobic PJI; 268 had aerobic PJI. Statistical analyses were performed using Pearson's $\chi^{2}$ , a Fisher exact test, and a t test. **Results**: Anaerobic PJIs represented 13.4 % of PJIs in our institution. Compared to aerobic cases, anaerobic PJIs had longer symptom duration (19.4 vs. 10.9 weeks, p=0.005 ), more sinus tracts (23.7 % vs. 6.1 %, p<0.001 ), fewer fevers (13.2 % vs. 31.3 %, p=0.022 ), more radiographic abnormalities (44.7 % vs. 29.3 %, p=0.024 ), and lower ESR and CRP (ESR: 49.0 vs. 67.4 mm h^−1^; CRP: 6.6 vs. 12.3 mg dL^−1^; both p=0.003 ). Shoulder PJIs were more often anaerobic (39.5 % vs. 4.9 %, p<0.001 ). Anaerobic PJIs were more likely to be treated with two-stage exchange (65.8 %), while aerobic cases more often underwent debridement and implant retention (44.7 %). Recurrence rates were similar. **Conclusion**: Anaerobic PJIs tend to present with features such as shoulder involvement, prolonged or chronic symptoms, sinus tract formation, and radiographic signs of infection, whereas aerobic PJIs are more commonly linked to acute presentations. For this reason, both aerobic and anaerobic cultures should be performed routinely to optimize diagnostic yield.

## 1 Introduction

Prosthetic joint infections (PJIs) are one of the most common complications after arthroplasties. Patients with PJIs experience higher overall mortality, greater use of assistive devices, reduced global quality of life, and higher costs compared to patients without PJI (Xu et al., 2023). PJIs occur in approximately 1 %–2 % of arthroplasties, a figure expected to rise with the growing demand for these procedures (Ahmed and Haddad, 2019; Tande and Patel, 2014). Gram-positive aerobic microorganisms, especially *Staphylococcus aureus* and coagulase-negative staphylococci, account for 50 %–60 % of cases, whereas aerobic Gram-negative bacilli account for less than 10 % (Tande and Patel, 2014). Anaerobic organisms, most notably *Cutibacterium acnes*, are identified in approximately 1 %–2 % of cases (Shah et al., 2015). Treatment guidelines do not routinely recommend empiric antibiotic coverage for anaerobic organisms (Osmon et al., 2013). However, with improved anaerobic bacteria isolation methods and the increased use of anaerobic cultures in suspected PJI cases in recent years (Yusuf et al., 2022), we hypothesized that the epidemiology of anaerobic PJIs may be evolving. This study aimed to characterize the clinical presentation, microbiology, and outcomes of anaerobic PJIs in comparison to those caused by aerobic organisms.

## 2 Methods

We conducted a retrospective chart review of patients aged 18 years and older who met Musculoskeletal Infection Society (MSIS) criteria (Parvizi et al., 2011) for PJI admitted to the University of Iowa Hospitals and Clinics (UIHC) from 2014 to 2020 who had a minimum of 3 months of follow-up from revision surgery. The year 2014 was selected as the starting point, as it marked the implementation of anaerobic cultures as a standard protocol for all patients with PJI. We identified 284 patients with PJI. The research protocol of this study was approved by our institutional review board with IRB number 202001293.

For each patient, intraoperative culture specimens were collected at the time of surgery using sterile techniques and stored in designated aerobic and anaerobic specimen collectors. Immediately after collection, the specimens were transported to the laboratory to ensure their viability. The specimens were processed using sterile disposable tissue grinders and were plated in a hood one sample at a time. Additionally, per institutional protocol, all patient specimens were sent for aerobic, anaerobic, fungal, and acid-fast bacilli (AFB) cultures. Species identification was performed using matrix-assisted laser desorption/ionization time-of-flight (MALDI-TOF) spectrometry.

Aerobic PJI was defined as PJI with aerobic organisms. On the other hand, anaerobic PJI was defined as PJI associated with anaerobic organisms or mixed organisms with anaerobes. We identified a total of 38 patients with anaerobic PJIs and 246 patients with aerobic PJIs. Demographic and clinical characteristics of the cohort were collected, including age, body mass index, other medical conditions, type of revision surgery performed, bacterial culture results, and recurrence of PJI. Descriptive and quantitative analyses were conducted where appropriate. For continuous variables of interest, the Student's t test was utilized. An alpha level of 0.05 was set as the threshold for significance. Additionally, logistic regression was employed to determine independent risk factors associated with anaerobic PJI.

## 3 Results

### 3.1 Demographics and clinical presentation

There were 38 (13.4 %) patients with anaerobic PJI and 246 (86.6 %) patients with aerobic PJI. The clinical characteristics of the patients are summarized in Table 1. The average age of patients in the anaerobic PJI cohort was 64.6 ( ±11.5 ) years and 65.9 ( ±11.1 ) years in the aerobic PJI cohort. The average BMIs were 32.9 ( ±6.1 ) and 34.3 ( ±8.8 ) for the anaerobic PJI and aerobic PJI cohorts, respectively. There were no statistical differences in the proportion of male patients in the anaerobic PJI (68.4 %) and aerobic PJI (52 %). There were also no statistical differences in the proportion of active smokers (21 % vs. 11 %) and alcohol users (42.1 % vs. 36.2 %) in the anaerobic and aerobic PJI cohorts. With regard to co-morbid conditions, the proportion of patients with chronic kidney disease was 15.8 % in the anaerobic PJI cohort and 13 % in the aerobic PJI cohort. The proportion of patients with chronic liver disease was 13.2 % and 7.4 % in the anaerobic and aerobic PJI groups, respectively.

**Table 1:** Baseline demographic and clinical characteristics of patients with anaerobic versus aerobic prosthetic joint infection (PJI).

	Anaerobic PJI ( n=38 )	Aerobic PJI ( n=246 )	P value
Average age (years)	64.6 ( ± 11.5)	65.9 ( ± 11.1)	0.496
Average body mass index	32.9 ( ± 6.1)	34.3 ( ± 8.8)	0.347
Male	26 (68.4 %)	128 (52 %)	0.059
Active smoker	8 (21 %)	27 (11 %)	0.910
Active alcohol user	16 (42.1 %)	89 (36.2 %)	0.585
Chronic kidney disease	6 (15.8 %)	32 (13 %)	0.639
Chronic liver disease	5 (13.2 %)	18 (7.4 %)	0.219
Diabetes mellitus	11 (28.9 %)	76 (30.9 %)	0.809
Fever	5 (13.2 %)	77 (31.3 %)	0.022
Joint pain	35 (92.1 %)	222 (90.2 %)	0.716
Joint swelling	20 (52.6 %)	157 (63.8 %)	0.185
Drainage	10 (26.3 %)	77 (31.3 %)	0.535
Joint warmth	1 (2.6 %)	48 (19.5 %)	0.010
Erythema	6 (15.8 %)	60 (24.4 %)	0.243
Presence of sinus tract	9 (23.7 %)	15 (6.1 %)	< 0.001
Duration of symptoms in weeks (weeks)	19.4 ± 15.7	10.6 ± 18.2	0.005
Shoulder PJI	15 (39.5 %)	12 (4.9 %)	< 0.001
Hip PJI	11 (28.9 %)	106 (43.1 %)	0.099
Knee PJI	12 (31.6 %)	131 (53.3 %)	0.013

Joint pain was the most common symptom followed by joint swelling in both groups. The proportion of patients with fever was higher in aerobic PJI (31.3 %) than in anaerobic PJI (13.2 %). Joint warmth was also more frequent in aerobic PJI (19.5 %) than in anaerobic PJI (2.6 %). On the other hand, sinus tract was also more common in the anaerobic PJI cohort (23.7 %) than in the aerobic PJI cohort (6.1 %). Duration of symptoms was also longer in anaerobic PJI (19.4 ± 15.7 weeks) than in aerobic PJI (10.6 ± 18.2 weeks). The differences between the two groups with regard to these characteristics reached statistical significance.

Anaerobic PJI (39.5 %) was statistically higher in prosthetic shoulder joints than aerobic PJI (4.9 %), largely driven by *C. acnes* infections. On the other hand, aerobic PJI (53.3 %) was more common than anaerobic PJI (31.6 %) in prosthetic knee joints, and the difference also reached statistical significance.

### 3.2 Radiographic and laboratory findings

Radiologic and lab findings are summarized in Table 2. Radiographic abnormalities were reported in a higher proportion of patients with anaerobic PJI (44.7 %) than aerobic PJI (29.3 %). Erythrocyte sedimentation rate (ESR) was higher in the aerobic PJI cohort (67.4 ± 36.0) than in the anaerobic PJI cohort (49 ± 27.5). C-reactive protein was also higher in the aerobic PJI cohort (12.3 ± 11.6) than in the anaerobic PJI cohort (6.6 ± 6.9). Bacteremia also occurred at a higher rate in the aerobic PJI cohort (19.9 %) than in the anaerobic PJI cohort (2.6 %). The differences for these reached statistical significance. The mean blood white blood cell count was 10.1 ± 5.3 in the anaerobic PJI cohort and 11.8 ± 7.7 in the aerobic PJI cohort. Additionally, the mean synovial white blood cell count was 44 329.3 ± 38 002 for the anaerobic PJI cohort and 67 994.9 ± 71 849.6 for the aerobic PJI cohort.

**Table 2:** Laboratory and radiographic characteristics of patients with anaerobic versus aerobic prosthetic joint infection (PJI).

	Anaerobic PJI ( n=38 )	Aerobic PJI ( n=246 )	P value
Presence of any radiographic abnormality	17 (44.7 %)	72 (29.3 %)	0.024
ESR (mm hg^−1^)	49 ± 27.5	67.4 ± 36.0	0.003
CRP (mg dL^−1^)	6.6 ± 6.9	12.3 ± 11.6	0.003
Blood white blood cell count	10.1 ± 5.3	11.8 ± 7.7	0.180
Synovial fluid white blood cell count	44 329.3 ± 38 002	67 994.9 ± 71 849.6	0.095
Presence of bacteremia	1 (2.6 %)	49 (19.9 %)	0.009

### 3.3 Microbiology of anaerobic PJI

Gram-positive organisms were the most commonly isolated organisms in our cohort (76.4 %), followed by anaerobes and polymicrobial cultures with anaerobes (13.4 %), Gram-negative organisms (7.7 %), and, finally, mixed aerobic cultures (2.5 %). The most common anaerobes isolated were *Cutibacterium* spp. (52 %), followed by *Finegoldia magna* (10 %), *Prevotella* spp. (8 %), *Actinomyces* spp. (6 %), *Anaerococcus* spp. (4 %), *Bacteroides fragilis* (4 %), *Peptoniphilus* spp. (2 %), *Paenibacillus* (1 %), *Peptostreptococcus anaerobius* (1 %), and *Porphyromonas* spp. (1 %) (Table 3). 

**Table 3:** Distribution of anaerobic organisms isolated in prosthetic joint infection (PJI).

Bacteria	Count (%)
*Cutibacterium* spp.	26 (52)
*Finegoldia magna*	5 (10)
*Prevotella* spp.	4 (8)
*Actinomyces* spp.	3 (6)
*Anaerococcus* spp.	2 (4)
*Bacteroides fragilis*	2 (4)
*Peptoniphilus* spp.	2 (4)
*Paenibacillus* spp.	1 (2)
*Peptostreptococcus anaerobius*	1 (2)
*Porphyromonas* spp.	1 (2)

**Figure 1:**
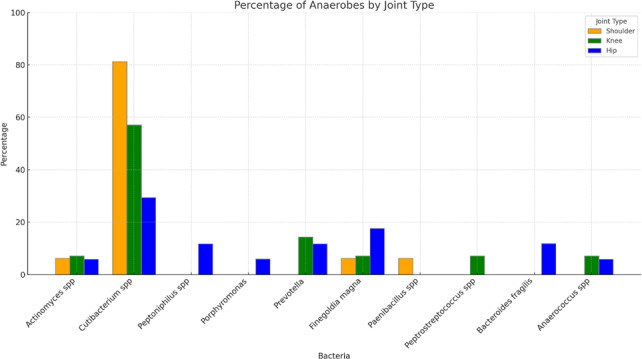
Distribution of different anaerobes in prosthetic joints of the shoulder, knee, and hip.

*Cutibacterium* spp. was the predominant anaerobe in all types of joints. It was found in 81.3 % (13 of 16) of anaerobic shoulder PJIs, 57.1 % of anaerobic knee PJIs, and 29.4 % of anaerobic hip PJIs. Non-*Cutibacterium* anaerobes were more diverse in hip and knee PJIs than in shoulder PJIs (Fig. 1).

### 3.4 Treatment and outcomes

The most common initial surgical management for all PJIs was two-stage exchange (50.4 %), followed by debridement and implant retention (DAIR) (41.5 %), one-stage exchange (6.3 %), joint aspiration only (1.1 %), and amputation (0.7 %). DAIR was more commonly performed for aerobic PJI (44.7 %) than for anaerobic PJI, and the difference was statistically significant. With regard to antibiotic treatment, unsurprisingly, anaerobic PJIs were treated more frequently with antibiotic regimens containing coverage for anaerobes (23.5 %) than for aerobic PJIs (4.1 %), and the difference was statistically significant. These antibiotics included metronidazole, ertapenem, ampicillin–sulbactam, and meropenem (Table 4).

**Table 4:** Surgical management strategies among patients with anaerobic versus aerobic prosthetic joint infection (PJI).

	Anaerobic PJI (%)	Aerobic PJI (%)	P value
DAIR	8 (21.1)	110 (44.7)	0.007
One-stage exchange	4 (10.5)	14 (5.7)	0.277
Two-stage exchange	25 (65.8)	118 (48.0)	0.054
Aspiration only	1 (2.6)	2 (0.8)	0.351
Amputation	0 (0)	2 (0.8)	1.000
Treatment with antibiotics covering anaerobes	12 (31.5)	11 (4.5)	< 0.001

**Table 5:** Clinical outcomes among patients with anaerobic versus aerobic prosthetic joint infection (PJI).

	Anaerobic PJI (%)	Aerobic PJI (%)	P value
No recurrence, on suppressive antibiotic	89	12	0.716
Resolution of infection (not on suppressive antibiotic)	109	16	0.862
Recurrence	38	5	1.000

Regarding outcomes, there were no significant differences between anaerobic and aerobic PJI in terms of recurrence rates, both on and off suppressive antibiotics, and in rates of resolution of infection (Table 5).

## 4 Discussion

PJIs remain a significant complication in orthopedic surgery. Historically, Gram-positive bacteria, especially staphylococci, are the predominant organisms that cause PJI, accounting for around 80 %–90 % of all PJI, whereas Gram-negative bacteria account for 11 % (Inman et al., 1984; Weinstein et al., 2023). Although data remain scarce, recent studies have highlighted the emerging significance of anaerobic bacteria and mixed infections in the etiology of PJIs (Lebowitz et al., 2017; Vajapey et al., 2021). Our retrospective, single-center cohort of adult patients with PJIs adds to this growing literature and underscores the need to consider anaerobes and polymicrobial infections in the diagnostic and therapeutic approach to PJI.

Our study identified a higher proportion of anaerobic PJIs (13.4 %) than Gram-negative PJIs (7.7 %), contrasting with previous reports that rank Gram-negative bacteria as the second most common cause of PJI (Weinstein et al., 2023; Pulido et al., 2008; Tsai et al., 2015). This rate also exceeds the 3 %–6 % typically cited in the literature (Tande and Patel, 2014; Shah et al., 2015). Several factors likely explain this discrepancy. Reporting practices vary, with some studies grouping anaerobes with Gram-positive or Gram-negative bacteria or reporting only monomicrobial cases. In addition, routine anaerobic cultures, prolonged incubation, and MALDI-TOF mass spectrometry at our institution likely enhanced detection. Literature estimates are further limited by inconsistent classification, with anaerobes often included in mixed infections or under Gram-negative bacilli (Liu et al., 2021). Anaerobes other than *C. acnes* are often found as part of polymicrobial infections. Notably, Marculescu and Cantey reported anaerobes in 11 % of PJIs overall, but with monomicrobial infections in only 2 %, consistent with our findings (Marculescu and Cantey, 2008).

A recent 2022 survey among clinical microbiologists in Europe revealed that up to 98 % of laboratories perform anaerobic cultures on all joint fluid samples suspected of PJI. This practice is likely paralleled in the United States, suggesting that detection rates of anaerobic organisms in PJIs may continue to rise and be reflected in future publications (Yusuf et al., 2022). A study by Triffault-Fillit et al. (2019) found a higher proportion of anaerobic PJIs (21.9 %) compared to Gram-negative PJIs (4.6 %), particularly in late-presenting PJIs (Triffault-Fillit et al., 2019). It is possible that Gram-negative bacteria are more common in early-onset infections in our cohort, although we did not stratify cases by infection acuity.

Our study confirmed that *Cutibacterium spp*. is the most frequently identified anaerobe across all joint types, corroborating recent research that highlights the significance of this bacterium in PJIs beyond shoulder arthroplasties (Karlsson et al., 2024; Reynolds et al., 2024). However, we observed a greater diversity of anaerobic species in knee and particularly in hip PJIs. In a study by Tsai et al. (2019), it was noted that there was a higher prevalence of polymicrobial organisms and anaerobes in hip PJIs when compared to knee infections (Tsai et al., 2019). These findings underscore the importance of obtaining anaerobic cultures from these joint types and suggest that broader empiric antibiotic coverage may be warranted for knee and hip PJIs, particularly when anaerobic pathogens are suspected.

With regard to clinical presentation, aerobic PJIs were more often associated with features of acute inflammation, such as fever, joint warmth, elevated inflammatory markers, and the presence of bacteremia. These findings are consistent with previous studies (Weinstein et al., 2023; Pulido et al., 2008; Tsai et al., 2015). In contrast, anaerobic PJIs were associated with more chronic signs of infection, like the presence of sinus tract, a longer duration of symptoms, and the presence of radiologic abnormalities. We postulate that the chronic/indolent nature of these infections may lead to longer time to diagnosis and that progressive radiologic abnormalities and sinus tracts might occur. Our results support the recent finding that polymicrobial PJIs are associated with chronic infection (Putnis et al., 2024).

The guidelines issued by the Infectious Diseases Society of America (IDSA) for managing PJIs recommend pathogen-specific antibiotic therapy with focus on Gram-positive organisms and select Gram-negative organisms. Among anaerobes, only *C. acnes* was specifically addressed (Osmon et al., 2013). In our study, *Cutibacterium* spp. was also the most frequently identified anaerobe, consistent with the prior literature (Triffault-Fillit et al., 2019). Other anaerobes, including *Finegoldia magna*, *Prevotella*, *Actinomyces*, *Anaerococcus*, and *Bacteroides fragilis*, may also contribute to PJI. Effective management requires pathogen-specific therapy guided by accurate microbiologic diagnosis. Future guidelines should better address the role of anaerobes and mixed flora in PJI and their implications for treatment.

In our cohort, anaerobic PJIs were managed differently compared to aerobic PJIs. There was a trend toward more frequent use of two-stage exchange procedures in cases of anaerobic infection. This is not unexpected, as two-stage exchange is typically preferred for late-presenting infections, which is where anaerobes and polymicrobial infections were most commonly encountered. Anaerobic PJIs were also more likely to be treated with antibiotic regimens that included anaerobic coverage. These variations in management may have contributed to the similar outcomes observed between anaerobic and aerobic PJIs (Vajapey et al., 2021).

This study exhibits several limitations that warrant consideration. Its single-center, retrospective design may limit the generalizability of the findings to other institutions or patient populations. Additionally, while the study observed no differences in outcomes between aerobic and anaerobic PJIs, it did not comprehensively evaluate how variations in management strategies between these groups may have influenced the results. Another limitation of this study is that inclusion required only a minimum of 3 months of follow-up after revision surgery. While most cases had longer follow-up, this threshold may not fully capture late recurrences that can occur in PJIs. These limitations underscore the need for further studies to improve the understanding and management of PJIs.

In conclusion, our study contributes to the literature on PJIs, particularly highlighting the role of anaerobes and mixed infections, which have historically been underreported in studies and clinical guidelines. Anaerobes may play a more important role in the etiology of PJIs than previously recognized. These findings support the routine inclusion of anaerobic cultures in diagnostic protocols for PJI and suggest that future guidelines should consider targeted management strategies for anaerobic infections.

## Data Availability

The datasets analyzed during the current study are not publicly available due to institutional data use agreements and patient privacy considerations but are available from the corresponding author upon reasonable request and with appropriate approvals.

## Code availability

Data were analyzed using IBM SPSS Statistics. The analyses were conducted using standard SPSS procedures. Syntax and output files are available from the corresponding author upon reasonable request.
